# Divergent Synthesis of Novel Five-Membered Heterocyclic Compounds by Base-Mediated Rearrangement of Acrylamides Derived from a Novel Isocyanide-Based Multicomponent Reaction

**DOI:** 10.3390/molecules16108775

**Published:** 2011-10-19

**Authors:** Andrea Basso, Luca Banfi, Renata Riva

**Affiliations:** Department of Chemistry and Industrial Chemistry, University of Genova, Via Dodecaneso 31, 16146 Genova, Italy

**Keywords:** multicomponent reactions, isocyanides, rearrangement reactions, diversity oriented synthesis

## Abstract

We have recently reported a novel multicomponent reaction between arylacetic acids and isocyanides, affording α-acyloxyacrylamides through an unusual mechanism. The products of this novel multicomponent reaction can rearrange to five membered heterocyclic compounds when exposed to an alkaline environment. Depending on the reaction conditions and on the substitution pattern on the substrates, various pyrrolidine derivatives can be selectively obtained. We now wish to report that libraries endowed with skeletal diversity, thus responding to the requirements of Diversity Oriented Synthesis (DOS), can be efficiently prepared in this manner, and phenotypic biological assays have shown interesting properties of some representative compounds.

## 1. Introduction

Isocyanide-based multicomponent reactions (I-MCRs) have received much attention during the last decades, not only for their ability to assemble in one step three or more building blocks in a single molecule but also because many heterocycles, relevant from a biological point of view, can be synthesised in a combinatorial fashion combining the multicomponent step with a post-condensation transformation [1,2].

**Scheme 1:**
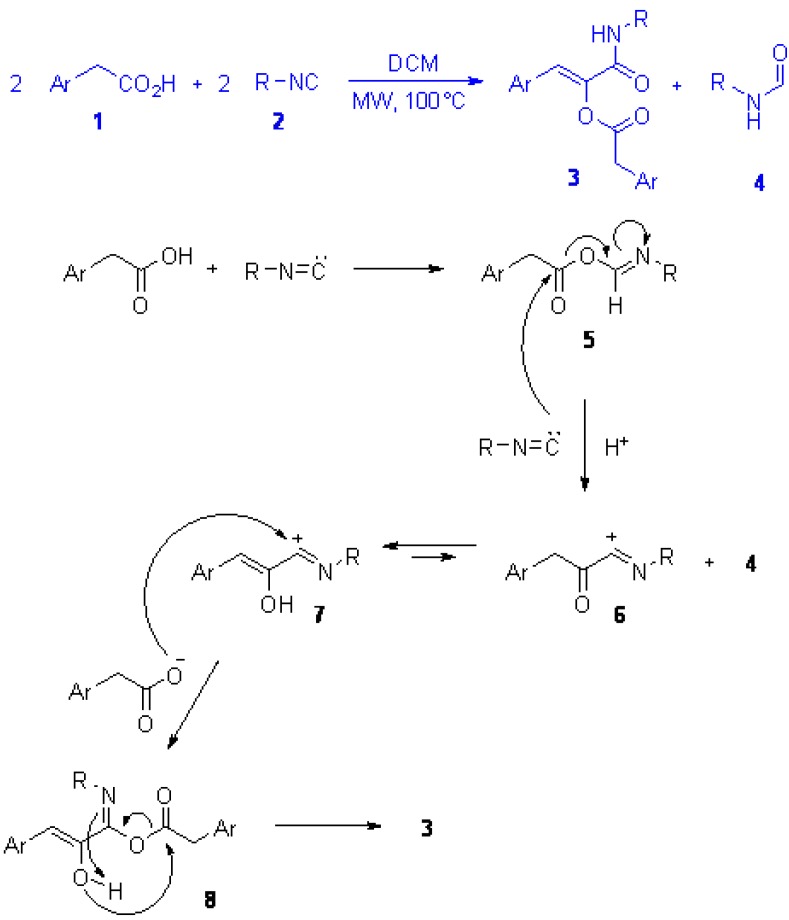
(**a**) The novel reaction between two molecules of arylacetic acid and two molecules of isocyanide, leading to α-acyloxyacrylamide **3** and formamide **4**; (**b**) Below, the postulated mechanism.

We have recently reported, in preliminary form, an unexpected reaction between arylacetic acids **1** and isocyanides **2**, leading to α-acyloxyacrylamides **3** (Scheme 1, top), and their rearrangement to give 5-membered heterocycles [3]. Although compounds **3** incorporate two molecules of the same carboxylic acid **1**, the reaction still falls under the definition of multicomponent condensation. In this full paper we report a more thorough discussion on this new methodology, as well as its application in the combinatorial synthesis of a library endowed with both scaffold and decoration diversity.

Scheme 1 (bottom) shows the postulated mechanism for the formation of **3**. Interaction of one molecule of acid with one of isocyanide forms an activated carboxylic acid ester **5** that reacts with one additional molecule of isocyanide to generate intermediate **6** that tautomerises to more stable **7** and is then attacked by a second molecule of acid to form **8**, finally rearranging to **3** in a way similar to the one occurring in the classic Passerini reaction. Thus, the reaction could be considered as a Passerini condensation where intermediate **5** behaves as a carbonyl surrogate, and intermediate **7** could be formally considered as the result of the addition of an isocyanide to an arylketene. To the best of our knowledge there is only one report where the carbonyl input is replaced in a Passerini reaction. Ugi early reported the reaction of diphenylketene with cyclohexylisocyanide in the presence of benzoic acid and the Passerini-like α-benzoyloxy-β,β-diphenylacrylamide was obtained as a consequence of the electrophilic addition of the ketene to the isocyanide, the former behaving as a carbonyl derivative [4]. Apart from this report, the reaction of ketenes with isocyanides and carboxylic acids has not been investigated further, probably due to the very limited number of stable ketenes available. Recently Pirali *et al.* have reported the reaction of acyl chlorides with α-isocyanoacetamides, leading to 2-acyl-5-aminooxazoles [5]; according to the postulated mechanism the ketene generated *in situ* from the acyl chloride in the presence of triethylamine reacts first with the isocyanide to form an acylnitrilium ion similar to **6**, that is subsequently trapped intramolecularly by the amidic oxigen to form the final oxazole.

**Figure 1:**
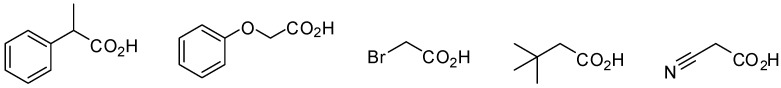
Carboxylic acids unable to furnish multicomponent adduct **3**.

Although in principle this reaction could be extended to any carboxylic acid, in practice we found that only arylacetic acids furnished compounds **3**, while other acids, like those reported in Figure 1, reacted with isocyanides under the same conditions to give mainly, via 1,3 O→N acyl shift of adduct **5**, *N*-formylamides as recently reported by Danishefsky [6]. A possible reason for this different behaviour can be associated with the stability of intermediate **7** where extensive conjugation with an aromatic ring is possible.

**Scheme 2:**
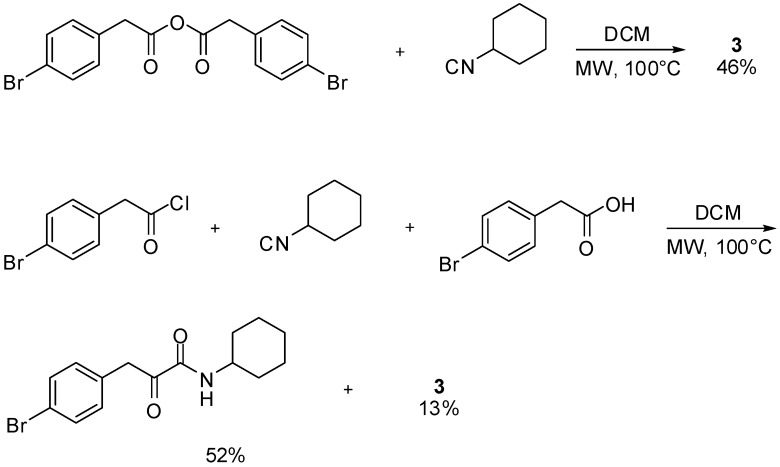
The reaction between activated carboxylic derivatives and isocyanides.

The novel reaction reported herein shows also the double reactivity of isocyanides: in the same reaction environment one molecule of isocyanide acts as an electrophile, activating the carboxylic acid, while another molecule acts as a nucleophile attacking the activated ester **5** to form **6**, and the secondary product **4**. Indeed, other activated carboxylic derivatives could replace adduct **5** and, for example, symmetrical anhydrides furnished compound **3** as well. In this case nucleophilic attack of the isocyanide onto one of the two carbonyls of the anhydride generated the second molecule of carboxylic acid required by the reaction. This strategy has the undoubtable advantage that the secondary formamide product **4** is not formed, thus requiring only one equivalent of isocyanide. However the need to prepare the symmetrical anhydride and the lower recovery yield of **3** convinced us to favour the synthetic route involving readily available arylacetic acids. In addition, arylacetyl chlorides also afforded compounds **3** in the presence of equimolar amounts of isocyanide and acid, although as minor component, since the main product was the ketoamide deriving from a Nef reaction (Scheme 2) [7].

## 2. Results and Discussion

**Scheme 3:**
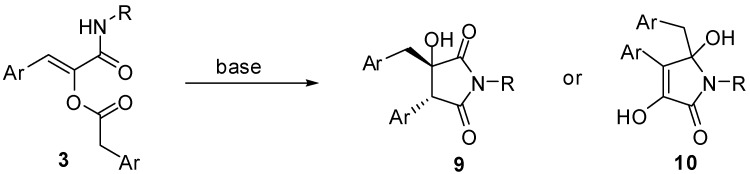
Products obtained by base-mediated rearrangement of compounds **3**.

A logical extension of this work would be to investigate whether it is possible to employ two different carboxylic acids and transform this reaction in a multicomponent condensation with three real diversity inputs. Although we are currently working on this challenging feature, we wish to report, in this paper, another interesting aspect of this reaction, that is the particular reactivity of α-acyloxyacrylamides. In fact, we have found that compounds **3** under basic conditions rearrange to 5-membered heterocycles. Specifically, pyrrolidine-2,5-diones **9** and pyrrolones **10** can be selectively obtained depending on the reaction conditions (Scheme 3) [3].

**Scheme 4:**
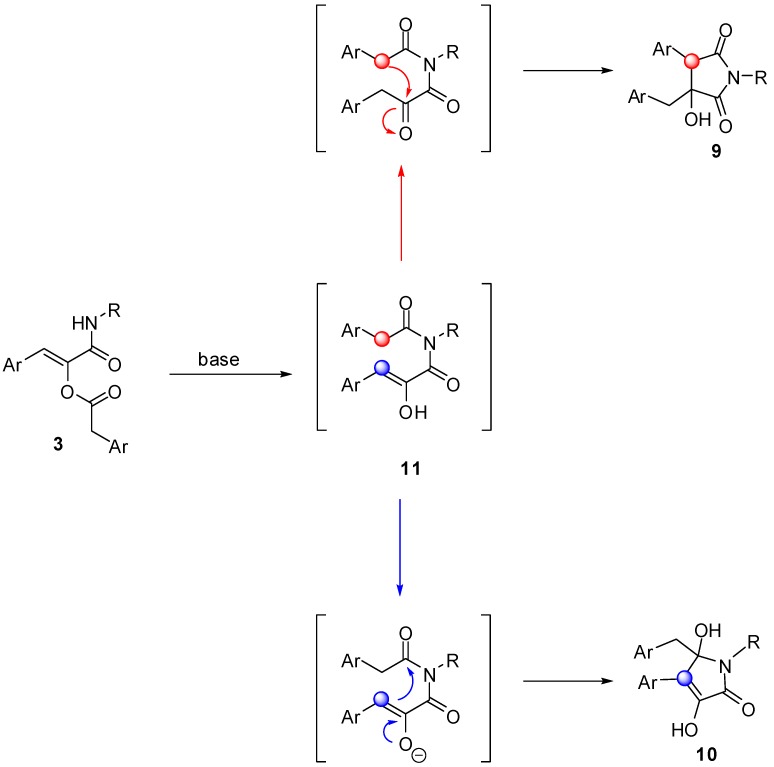
Postulated mechanism for the formation of heterocycles **9** and **10**.

Intrigued by this outcome, we have tried to rationalise it by postulating that in the presence of a base, the first event to occur is migration of the acyl group of **3** onto the amidic nitrogen, forming an imide derivative **11**. In the case of classic Passerini adducts, this intermediate is highly unstable and spontaneously hydrolyses liberating the corresponding α-hydroxyamide. On the other hand, imides generated from **3**, although never isolated, can further evolve via an intramolecular aldol-like reaction, involving as nucleophile either the methylene of the arylacetyl group (red) or the enol (blue) liberated by the acyl migration. In the former case compound **9** is generated after enol tautomerisation, while in the latter it is **10** that would be formed, presumably after enol deprotonation (Scheme 4). Indeed, using a weak base (triethylamine) under microwave heating, pyrrolidine-2,5-diones **9** are obtained exclusively, while using stronger bases (sodium or potassium *tert*-butoxide or sodium hydride) pyrrolone derivatives **10** are generated immediately, even at room temperature.

The exclusive formation of **9** or **10** depending on the reaction conditions, prompted us to investigate whether factors other than the strengh of the base could influence the outcome. In particular, we have studied whether the counterion of the strong base could have an effect, for example by directing the functional groups involved in the cyclisation in close proximity. For this purpose we used LiHMDS as a base with the idea that, Li^+^ being able to form strong chelates, a different outcome could be observed. However, compound **10** was again isolated in comparable yield and purity. In another experiment we investigated the effect of the solvent, by performing the reaction with Et_3_N in *t*-BuOH, the solvent generally used to obtain **10**, but compound **9** was obtained regularly. In order to determine the amount of base required by the two reactions, two experiments were run under the standard conditions used to obtain **9** and **10**, but with only 0.1 equivalents of Et_3_N and *t*-BuONa respectively. In the former case the final product was isolated quantitatively, while in the latter only 10% conversion was observed. These experiments confirmed that, when the base employed is strong enough to quantitatively generate the enolate, the path followed is the one leading to **10**, while when the reaction conditions allow for the tautomerisation of **11**, this reacts selectively to give **9**.

In the case of compound **9** one of the two possible diastereoisomers is formed selectively, and early n.O.e. experiments served to determine that the phenyl and benzyl substituents are in relative *anti* configuration.

**Scheme 5:**
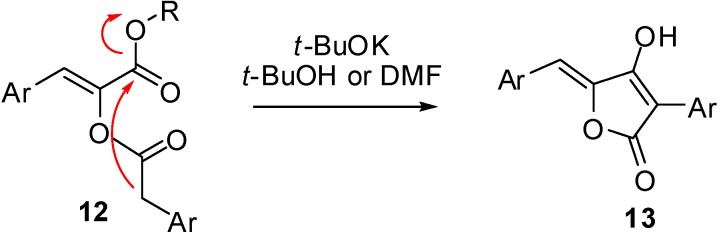
Synthesis of pulvinones through Dieckmann condensation of α-arylacetyloxy crylesters.

To the best of our knowledge the reactivity of **3** under basic conditions was unprecedented in the literature, the only result that at first sight could resemble our findings was a report by Brückner on the synthesis of pulvinones [8]. Compounds **12**, structurally very similar to **3**, afforded, upon treatment with *t*-BuOK in *t*-BuOH or DMF, compounds **13**; however a closer look revealed that in this case a Dieckmann condensation occurred, that in our case would not have been possible due to the higher acidity of the amidic hydrogen compared to the ester enolate. Moreover, compounds **13** were generally obtained under harsher conditions (3 equivalents of base at reflux for 1–2 h) and in lower yields (Scheme 5).

Although we were expecting that bulky substituents onto the nitrogen atom of **3** could prevent the reactions to occur, we were surprised to find that derivatives with a *N*-cyclohexyl substituent smoothly furnished compound **9**, but yielded only traces of product **10**, along with large amount of ketoamide resulting from hydrolysis. The reason for this strange behaviour was attributed not to a different reactivity but rather to the instability of compounds **10** with a *N*-cyclohexyl substituent: indeed they were found to be unstable upon storage, furnishing the open adducts that then would hydrolyse. A different result was obtained with *N*-*t*-butyl amides: In this case, as expected, the acyl migration did not occur due to bulkiness of the N-substituent, and starting material was recovered unreacted.

The possibility of elaborating a substrate in many distinct ways depending on the reaction conditions is a requirement for pluripotent substrates, very versatile starting materials in diversity oriented synthesis [9]. Indeed α-acyloxyacrylamides can generate two distinct classes of nitrogen heterocycles depending on the conditions employed during the rearrangement step: with these results in hand we decided to prepare a pivotal library of compounds **3**, **9** and **10** employing three different arylacetic acids and isocyanides. The power of this approach is that large libraries of original structures can be obtained with little effort: by employing only six different building blocks a library of 27 compounds can be obtained with three diverse skeletons and nine diverse appendage combinations. Moreover, the library synthesis was facilitated by the fact that the complex heterocyclic structures of **9** and **10** could be assembled in just two synthetic steps from the starting materials and that most of the products could be isolated in pure form by simple precipitation from the crude. Table 1 summarises the results obtained.

It is interesting to note that in the case of 4-chlorophenylacetic acid (Table 1, entries b, e and h), the rearrangement to pyrrolidinedione **9** had to be performed with a catalytic amount of Et_3_N. When larger quantities were employed, elimination of water was observed, with subsequent formation of a mixture of dehydration products with *exo* and *endo* cyclic double bonds.

**Table 1:** Results obtained for the synthesis of the 27 member library employing three different arylacetic acids and three different isocyanides.

Entry	Isocyanide	Acid	Yield (3)	Yield (9)	Yield (10)
**a**	*n*-butyl	phenylacetic	90%	54%	65%
**b**	*n*-butyl	4-Cl-phenylacetic	58%	78% ^a^	65%
**c**	*n*-butyl	3-MeO-phenylacetic	70%	76%	67%
**d**	benzyl	phenylacetic	77%	65%	72%
**e**	benzyl	4-Cl-phenylacetic	64%	69%^a^	72%
**f**	benzyl	3-MeO-phenylacetic	76%	78%	56%
**g**	BnOCO-ethyl	phenylacetic	76%	65%	78%
**h**	BnOCO-ethyl	4-Cl-phenylacetic	54%	75% ^a^	75%
**i**	BnOCO-ethyl	3-MeO-phenylacetic	83%	72%	76%

^a^ Reactions performed with 0.1 equivalents of Et_3_N.

**Figure 2:**
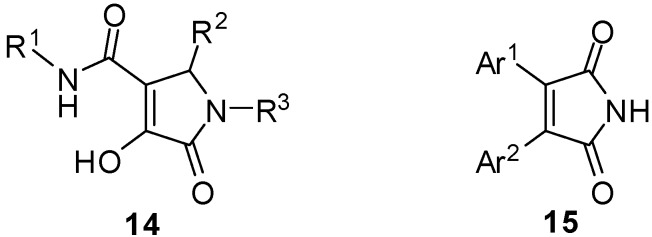
Molecules structurally related to **10** and **9** showing biological activity.

There are not many biological data on compounds structurally similar to **9** and **10**, for example compounds of general formula **14** have recently found biological applications as HIV-1 integrase inhibitors [10], while compounds **15** (Figure 2) have shown activity as PGE_2_ production inhibitors [11].

Intrigued by the rather unique substitution pattern shown by derivatives **9** and **10**, we subjected the library members to phenotypic assays, following the PD^2^ project set up by Eli Lilly [12]. Our intention was to also submit compounds **3**, but these were rejected by the software, being recognised as potential alkylating agents (however, in our experience, such compounds react very poorly with nucleophiles such as amines or thiols). We were pleased that some pyrrolidine-2,5-dione derivatives were found to be active in both anti-angiogenesis and diabetes phenotypic assays; in particular, compound **9b** inhibited angiogenesis in human endotelial progenitor cells (ECFCs) with an IC_50_ of 5.2 μM, without inhibition of known angiogenesis receptor tyrosine kinases. We are currently trying to modify the structure of such hits in order to improve activity and solubility. These results will be reported in due course.

## 3. Experimental

### 3.1. General

^1^H-NMR and ^13^C-NMRs were recorded on a Varian MERCURYplus 300 instrument at 300/75 MHz. Chemical shifts are reported in p.p.m. using TMS (0.00 ppm) as internal standard for ^1^H spectra and the residual solvent peak for ^13^C ones. Coupling constants (*J*) are reported in Hz. HRMS were recorded with a Micromass AutoSpec instrument at the Parque Cientifico Tecnologico of the University of Burgos (Spain). Microwave heating was performed in a CEM Discover apparatus equipped with infrared detector for temperature measurement. All reagents have been bought from Fluka or Aldrich.

### 3.2. General Procedure for the Synthesis of Compounds **3**

The acid (1 mmol) and the isocyanide (1 mmol) were dissolved in dry dichloromethane (DCM, 1 mL) in a closed MW vessel and heated at 100 °C for 30 min using 150 W power. The resulting mixture was then diluted with DCM (9 mL) and washed with saturated sodium bicarbonate (2 × 5 mL). The organic layer was dried with sodium sulphate, filtered, concentrated *in vacuo* and purified by flash chromatography with PE/EtOAc eluent.

*(Z)-3-(Butylamino)-3-oxo-1-phenylprop-1-en-2-yl 2-phenylacetate* (**3a**). ^1^H-NMR (CDCl_3_) δ 0.87 (t, *J* = 7.1 Hz, 3H), 1.15–1.33 (m, 4H), 3.13 (q, *J* = 6.9 Hz, 2H), 3.83 (s, 2H), 5.61 (br s, 1H), 7.22–7.33 (m, 5H), 7.34–7.46 (m, 6H); ^13^C-NMR (CDCl_3_) δ 168.01, 162.47, 139.46, 132.87, 132.51, 129.68, 129.57, 129.34, 129.28, 128.82, 128.08, 124.20, 41.89, 39.59, 31.46, 20.07, 13.89; HRMS: calcd. for C_21_H_23_NO_3_ 337.1678, found 337.1670.

*(Z)-3-(Butylamino)-1-(4-chlorophenyl)-3-oxoprop-1-en-2-yl 2-(4-chlorophenyl)acetate* (**3b**). ^1^H-NMR (CDCl_3_) δ 0.90 (t, *J* = 7.1 Hz, 3H), 1.15–1.35 (m, 4H), 3.20 (q, *J* = 6.9 Hz, 2H), 3.80 (s, 2H), 5.53 (br s, 1H), 7.10–7.40 (m, 9H); ^13^C-NMR (CDCl_3_) δ 167.35, 161.93, 139.79, 135.04, 134.10, 130.89, 130.74, 130.65, 130.57, 129.32, 128.92, 122.61, 40.90, 39.50, 31.34, 19.95, 13.72; HRMS: calcd. for C_21_H_21_Cl_2_NO_3_ 405.0898, found 405.0892.

*(Z)-3-(Butylamino)-1-(3-methoxyphenyl)-3-oxoprop-1-en-2-yl 2-(3-methoxyphenyl)acetate* (**3c**). ^1^H-NMR (CDCl_3_) δ 0.87 (t, *J* = 7.0 Hz, 3H), 1.12–1.30 (m, 4H), 3.12 (q, *J* = 6.6 Hz, 2H), 3.79 (s, 3H), 3.80 (br s, 5H), 5.53 (t, *J* = 5.3 Hz, 1H), 6.80–7.05 (m, 6H), 7.15–7.35 (m, 3H); ^13^C-NMR (CDCl_3_) δ 167.83, 162.26, 160.34, 159.75, 139.51, 134.25, 133.79, 130.41, 129.85, 124.30, 122.07, 121.65, 115.23, 115.03, 114.94, 113.61, 55.45, 55.39, 41.96, 39.60, 31.45, 20.10, 13.92; HRMS: calcd. for C_23_H_27_NO_5_ 397.1889, found 397.1891.

*(Z)-3-(Benzylamino)-3-oxo-1-phenylprop-1-en-2-yl 2-phenylacetate* (**3d**). ^1^H-NMR (CDCl_3_) δ 3.81 (s, 2H), 4.35 (d, *J* = 5.9 Hz, 2H), 5.81 (br s, 1H), 7.10–7.40 (m, 16H); ^13^C-NMR (CDCl_3_) δ 168.06, 162.47, 139.26, 137.84, 132.64, 132.43, 129.77, 129.44, 129.29, 128.89, 128.88, 128.06, 127.92, 127.75, 124.73, 43.82, 41.92 (two C-H aromatic signals overlapped); HRMS: calcd. for C_24_H_21_NO_3_ 371.1521, found 371.1540.

*(Z)-3-(Benzylamino)-1-(4-chlorophenyl)-3-oxoprop-1-en-2-yl 2-(4-chlorophenyl)acetate* (**3e**). ^1^H-NMR (CDCl_3_) δ 3.75 (s, 2H), 4.38 (d, *J* = 5.7 Hz, 2H), 6.13 (t, *J* = 5.7 Hz, 1H), 7.10–7.35 (m, 14H); ^13^C-NMR (CDCl_3_) δ 167.45, 161.96, 139.53, 137.40, 135.19, 133.96, 130.64, 130.57, 130.53, 129.26, 128.95, 128.81, 127.77, 123.18, 43.73, 40.85 (two C-H aromatic signals overlapped); HRMS: calcd. for C_24_H_19_Cl_2_NO_3_ 439.0742, found 439.0740.

*(Z)-3-(Benzylamino)-1-(3-methoxyphenyl)-3-oxoprop-1-en-2-yl 2-(3-methoxyphenyl)acetate* (**3f**). ^1^H-NMR (CDCl_3_) δ 3.69 (s, 3H), 3.76 (br s, 5H), 4.32 (d, *J* = 5.9 Hz, 2H), 5.97 (t, *J* = 5.8 Hz, 1H), 6.70–7.30 (m, 14H); ^13^C-NMR (CDCl_3_) δ 167.99, 162.40, 160.17, 159.73, 139.39, 137.83, 134.01, 133.64, 130.24, 129.84, 128.79, 127.70, 127.60, 124.71, 122.10, 121.44, 115.28, 115.04, 114.93, 113.58, 55.36, 55.33, 43.70, 41.82; HRMS: calcd. for C_26_H_25_NO_5_ 431.1733, found 431.1715.

*(Z)-Benzyl 3-(3-phenyl-2-(2-phenylacetoxy)acrylamido)propanoate* (**3g**). ^1^H-NMR (CDCl_3_) δ 2.57 (t, *J* = 6.0 Hz, 2H), 3.54 (q, *J* = 6.1 Hz, 2H), 3.83 (s, 2H), 5.13 (s, 2H), 6.50 (br s, 1H), 7.12–7.49 (m, 16H); ^13^C NMR (CDCl_3_) δ 172.72, 168.28, 162.55, 139.49, 135.72, 132.67, 132.33, 129.80, 129.77, 129.35, 129.20, 128.86, 128.80, 128.62, 128.44, 127.96, 124.24, 66.84, 41.61, 35.25, 33.81; HRMS: calcd. for C_27_H_25_NO_5_ 443.1733, found 443.1740.

*(Z)-Benzyl 3-(3-(4-chlorophenyl)-2-(2-(4-chlorophenyl)acetoxy)acrylamido)propanoate* (**3h**). ^1^H-NMR (CDCl_3_) δ 2.61 (t, *J* = 6.0 Hz, 2H), 3.58 (q, *J* = 6.0 Hz, 2H), 3.79 (s, 2H), 5.13 (s, 2H), 6.68 (t, *J* = 6.0 Hz, 1H), 7.11 (s, 1H), 7.19 (s, 5H), 7.23–7.41 (m, 8H); ^13^C-NMR (CDCl_3_) δ 172.96, 167.87, 162.20, 140.00, 135.62, 135.29, 134.05, 131.10, 130.95, 130.86, 130.69, 129.33, 129.04, 128.88, 128.66, 128.40, 122.91, 66.90, 40.75, 35.22, 33.70; HRMS: calcd. for C_27_H_23_Cl_2_NO_5_ 511.0953, found 511.1001.

*(Z)-Benzyl 3-(3-(3-methoxyphenyl)-2-(2-(3-methoxyphenyl)acetoxy)acrylamido)propanoate* (**3i**). ^1^H-NMR (CDCl_3_) δ 2.55 (t, *J* = 6.1 Hz, 2H), 3.52 (q, *J* = 6.3 Hz, 2H), 3.76 (s, 3H), 3.77 (s, 3H), 3.81 (s, 2H), 5.12 (s, 2H), 6.46 (t, *J* = 5.9 Hz, 1H), 6.81–6.99 (m, 6H), 7.16 (t, *J* = 8.0 Hz, 1H), 7.21 (s, 1H), 7.24–7.40 (m, 6H); ^13^C-NMR (CDCl_3_) δ 172.59, 168.20, 162.44, 160.17, 159.72, 139.61, 135.72, 134.01, 133.60, 130.19, 129.81, 128.83, 128.60, 128.44, 124.30, 122.10, 121.90, 115.37, 115.25, 115.02, 113.44, 66.82, 55.42, 55.39, 41.53, 35.27, 33.77; HRMS: calcd. for C_29_H_29_NO_7_ 503.1944, found 503.1958.

### 3.3. General Procedure for the Synthesis of Compounds **9**

Compound **3** (0.5 mmol) and triethylamine (0.5 mmol) were dissolved in dry benzene (1 mL) in a closed MW vessel and heated at 90 °C for 30 min with 100 W power. The resulting suspension was diluted with Et_2_O (2 mL) and filtered. The solid was then washed with PE/Et_2_O 1:1 (5 mL) and dried under vacuum or alternatively was purified by flash chromatography with PE/EtOAc eluent.

*3-Benzyl-1-butyl-3-hydroxy-4-phenylpyrrolidine-2,5-dione* (**9a**). ^1^H-NMR (CDCl_3_) δ 0.91 (t, *J* = 7.2 Hz, 3H), 1.18–1.33 (m, 2H), 1.48–1.60 (m, 2H), 2.60–2.90 (br s, 1H), 3.09 (d, *J* = 13.5 Hz, 1H), 3.26 (d, *J* = 13.5 Hz, 1H), 3.53 (t, *J* = 7.3 Hz, 2H), 4.05 (s, 1H), 6.90–6.98 (m, 2H), 7.25–7.38 (m, 8H); ^13^C-NMR (CDCl_3_) δ 178.61, 175.65, 134.29, 132.18, 130.70, 129.97, 129.08, 129.02, 128.48, 127.93, 77.14, 54.62, 44.18, 39.09, 29.82, 20.18, 13.74; HRMS: calcd. for C_21_H_23_NO_3_ 337.1678, found 337.1670.

*1-Butyl-3-(4-chlorobenzyl)-4-(4-chlorophenyl)-3-hydroxypyrrolidine-2,5-dione* (**9b**). ^1^H-NMR (CDCl_3_) δ 0.92 (t, *J* = 7.2 Hz, 3H), 1.18–1.32 (m, 2H), 1.47–1.60 (m, 2H), 2.84 (s, 1H), 3.04 (d, *J* = 13.7 Hz, 1H), 3.19 (d, *J* = 13.7 Hz, 1H), 3.54 (t, *J* = 7.3 Hz, 2H), 3.94 (s, 1H), 6.88 (d, *J* = 8.4 Hz, 2H), 7.19 (d, *J* = 8.4 Hz, 2H), 7.25–7.35 (m, 4H); ^13^C-NMR (CDCl_3_) δ 178.50, 175.31, 134.57, 134.10, 132.49, 131.95, 131.36, 130.32, 129.27, 129.12, 53.78, 43.12, 39.21, 29.77, 20.14, 13.77 (*one signal covered by solvent residual peak*); HRMS: calcd. for C_21_H_21_Cl_2_NO_3_ 405.0898, found 405.0887.

*1-Butyl-3-hydroxy-3-(3-methoxybenzyl)-4-(3-methoxyphenyl)pyrrolidine-2,5-dione* (**9c**). ^1^H-NMR (CDCl_3_) δ 0.92 (t, *J* = 7.3 Hz, 3H), 1.20–1.35 (m, 2H), 1.48–1.63 (m, 2H), 2.43 (s, 1H), 3.07 (d, *J* = 13.5 Hz, 1H), 3.23 (d, *J* = 13.5 Hz, 1H), 3.55 (t, *J* = 7.4 Hz, 2H), 3.73 (s, 3H), 3.78 (s, 3H), 4.05 (s, 1H), 6.40–6.60 (m, 2H), 6.75–6.95 (m, 4H), 7.20–7.30 (m, 2H); ^13^C-NMR (CDCl_3_) δ 178.47, 175.57, 160.05, 159.96, 135.70, 133.34, 130.12, 130.08, 122.89, 121.94, 116.46, 115.88, 113.95, 113.15, 77.02, 55.38, 55.34, 54.36, 43.94, 39.09, 29.81, 20.17, 13.79; HRMS: calcd. for C_23_H_27_NO_5_ 397.1889, found 397.1871.

*1,3-Dibenzyl-3-hydroxy-4-phenylpyrrolidine-2,5-dione* (**9d**). ^1^H-NMR (CDCl_3_) δ 3.08 (d, *J* = 13.5 Hz, 1H), 3.26 (d, *J* = 13.5 Hz, 1H), 4.06 (s, 1H), 4.67 (d, *J* = 14.1 Hz, 1H), 4.73 (d, *J* = 14.1 Hz, 1H), 5.30 (s, 1H), 6.80–6.94 (m, 2H), 7.12–7.42 (m, 13H); ^13^C-NMR (CDCl_3_) δ 178.05, 175.32, 135.43, 134.01, 131.86, 130.63, 129.88, 129.12, 128.95, 128.84, 128.62, 128.30, 127.94, 54.61, 44.02, 42.86 (*two C–H aromatic signals overlapped*); HRMS: calcd. for C_24_H_21_NO_3_ 371.1521, found 371.1508.

*1-Benzyl-3-(4-chlorobenzyl)-4-(4-chlorophenyl)-3-hydroxypyrrolidine-2,5-dione* (**9e**). ^1^H-NMR (CDCl_3_) δ ^1^H-NMR (300 MHz, CDCl_3_) δ 2.73 (s, 1H), 2.99 (d, *J* = 13.7 Hz, 1H), 3.17 (d, *J* = 13.7 Hz, 1H), 3.94 (s, 1H), 4.66 (d, *J* = 13.8 Hz, 1H), 4.72 (d, *J* = 13.8 Hz, 1H), 6.82 (d, *J* = 8.4 Hz, 2H), 7.06 (d, *J* = 8.4 Hz, 2H), 7.15–7.35 (m, 9H); ^13^C-NMR (CDCl_3_) δ 178.05, 174.88, 135.14, 134.62, 134.00, 132.22, 131.83, 131.28, 130.24, 129.25, 129.15, 128.99, 128.79, 128.47, 76.91, 53.98, 43.18, 42.93; HRMS: calcd. for C_24_H_19_Cl_2_NO_3_ 439.0742, found 439.0737.

*1-Benzyl-3-hydroxy-3-(3-methoxybenzyl)-4-(3-methoxyphenyl)pyrrolidine-2,5-dione* (**9f**). ^1^H-NMR (CDCl_3_) δ 2.22 (s, 1H), 3.07 (d, *J* = 13.5 Hz, 1H), 3.21 (d, *J* = 13.5 Hz, 1H), 3.64 (s, 3H), 3.73 (s, 3H), 4.07 (s, 1H), 4.68 (d, *J* = 14.1 Hz, 1H), 4.74 (d, *J* = 14.1 Hz, 1H), 6.33–6.37 (m, 1H), 6.46 (d, *J* = 7.6 Hz, 1H), 6.76–6.86 (m, 4H), 7.15–7.24 (m, 2H), 7.27–7.38 (m, 5H); ^13^C-NMR (CDCl_3_) δ 177.98, 175.17, 160.06, 160.02, 135.57, 135.46, 133.18, 130.20, 130.12, 128.96, 128.79, 128.26, 122.85, 121.99, 116.34, 115.28, 114.39, 113.33, 55.39, 55.30, 54.56, 43.92, 42.85 (aliphatic quaternary C signal covered by residual solvent peak); HRMS: calcd. for C_26_H_25_NO_5_ 431.1733, found 431.1731.

*Benzyl 3-(3-benzyl-3-hydroxy-2,5-dioxo-4-phenylpyrrolidin-1-yl)propanoate* (**9g**). ^1^H-NMR (CDCl_3_) δ 2.24 (s, 1H), 2.68 (t, *J* = 7.2 Hz, 2H), 3.11 (d, *J* = 13.5 Hz, 1H), 3.21 (d, *J* = 13.5 Hz, 1H), 3.79–3.95 (m, 2H), 4.05 (s, 1H), 5.11 (s, 2H), 6.87–6.95 (m, 2H), 7.22–7.40 (m, 13H); ^13^C-NMR (CDCl_3_) δ 178.16, 175.37, 170.59, 135.55, 134.11, 131.78, 130.64, 129.90, 129.08, 129.05, 128.78, 128.66, 128.61, 128.54, 127.95, 77.08, 67.05, 54.59, 43.84, 34.93, 32.04; HRMS: calcd. for C_27_H_25_NO_5_ 443.1733, found 443.1718.

*Benzyl 3-(3-(4-chlorobenzyl)-4-(4-chlorophenyl)-3-hydroxy-2,5-dioxopyrrolidin-1-yl)propanoate* (**9h**). ^1^H-NMR (CDCl_3_) δ 2.67 (s, 1H), 2.70 (t, *J* = 6.6 Hz, 2H), 3.03 (d, *J* = 13.8 Hz, 1H), 3.13 (d, *J* = 13.8 Hz, 1H), 3.80–3.95 (m, 3H), 5.09 (s, 2H), 6.84 (dm, *J* = 8.4 Hz, 2H), 7.17 (dm, *J* = 8.4 Hz, 2H), 7.25 (dm, *J* = 8.4 Hz, 2H), 7.31 (dm, *J* = 8.4 Hz, 2H), 7.33–7.38 (m, 5H); ^13^C-NMR (CDCl_3_) δ 177.91, 174.99, 170.67, 135.44, 134.62, 134.07, 132.51, 132.00, 131.38, 130.07, 129.33, 129.26, 129.14, 128.83, 128.71, 67.20, 53.98, 42.85, 35.15, 31.95 (aliphatic quaternary C signal covered by residual solvent peak); HRMS: calcd. for C_27_H_23_Cl_2_NO_5_ 511.0953, found 511.0938.

*Benzyl 3-(3-hydroxy-3-(3-methoxybenzyl)-4-(3-methoxyphenyl)-2,5-dioxopyrrolidin-1-yl)propano-ate* (**9i**). ^1^H-NMR (CDCl_3_) δ 2.18 (s, 1H), 2.69 (td, *J* = 7.1, 2.3 Hz, 2H), 3.09 (d, *J* = 13.5 Hz, 1H), 3.17 (d, *J* = 13.5 Hz, 1H), 3.73 (s, 3H), 3.78 (s, 3H), 3.88 (t, *J* = 6.9 Hz, 2H), 4.05 (s, 1H), 5.11 (s, 2H), 6.42–6.45 (m, 1H), 6.52 (d, *J* = 7.5 Hz, 1H), 6.77–6.86 (m, 4H), 7.15–7.30 (m, 2H), 7.32–7.38 (m, 5H); ^13^C-NMR (CDCl_3_) δ 178.00, 175.17, 170.62, 160.09, 160.07, 135.64, 135.61, 133.10, 130.26, 130.14, 128.82, 128.70, 128.65, 122.91, 121.83, 116.57, 115.81, 114.16, 113.20, 77.00, 67.10, 55.44, 55.41, 54.66, 43.91, 35.02, 32.07; HRMS: calcd. for C_29_H_29_NO_7_ 503.1944, found 503.1933.

### 3.4. General Procedure for the Synthesis of Compounds **10**

Compound **3** (0.5 mmol) was suspended in dry *t*-BuOH (2 mL) and sodium *t*-butoxide (0.55 mmol) was added; the solution turned yellow immediately and a TLC control showed complete disappearance of starting material. The reaction was quenched with 0.5 M NH_4_H_2_PO_4_ (5 mL) and extracted with Et_2_O (2 × 5 mL). The combined organics were anhydrified with sodium sulphate, filtered and concentrated in vacuo. The crude was taken up in DCM (3 mL), a white/yellow solid crashed down and it was collected by filtration. Alternatively the crude was purified by flash chromatography with PE/EtOAc eluent.

*5-Benzyl-1-butyl-3,5-dihydroxy-4-phenyl-1H-pyrrol-2(5H)-one* (**10a**). ^1^H-NMR (DMSO-*d_6_*) δ 0.93 (t, *J* = 7.2 Hz, 3H), 1.27–1.43 (m, 2H), 1.57–1.75 (m, 2H), 3.19 (d, *J* = 13.8 Hz, 1H), 3.28 (d, *J* = 13.7 Hz, 1H), 3.32–3.47 (m, 2H), 6.50 (s, 1H), 6.69 (dd, *J* = 6.6, 2.9 Hz, 2H), 7.01–7.12 (m, 3H), 7.28 (t, *J* = 7.2 Hz, 1H), 7.44 (t, *J* = 7.8 Hz, 2H), 8.01 (dd, *J* = 8.4, 1.0 Hz, 2H), 10.02 (s, 1H); ^13^C-NMR (DMSO-*d_6_*) δ 164.70, 144.39, 135.54, 132.89, 129.61, 128.63, 127.87, 127.69, 127.05, 126.84, 118.97, 90.78, 41.30, 39.09, 31.09, 20.36, 14.10; HRMS: calcd. for C_21_H_23_NO_3_ 337.1678, found 337.1690.

*1-Butyl-5-(4-chlorobenzyl)-4-(4-chlorophenyl)-3,5-dihydroxy-1H-pyrrol-2(5H)-one* (**10b**). ^1^H-NMR (DMSO-*d_6_*) δ 0.92 (t, *J* = 7.3 Hz, 3H), 1.27–1.42 (m, 2H), 1.55–1.74 (m, 2H), 3.14 (d, *J* = 13.8 Hz, 1H), 3.30 (d, *J* = 13.8 Hz, 1H), 3.39 (t, *J* = 7.2 Hz, 2H), 6.61 (s, 1H), 6.68 (d, *J* = 8.4 Hz, 2H), 7.15 (d, *J* = 8.3 Hz, 2H), 7.51 (d, *J* = 8.7 Hz, 2H), 7.99 (d, *J* = 8.7 Hz, 2H), 10.35 (s, 1H); ^13^C-NMR (DMSO-*d_6_*) δ 164.08, 145.04, 134.27, 131.40, 131.21, 131.09, 130.91, 128.92, 128.43, 127.55, 117.29, 90.18, 30.82, 20.06, 13.80 (*two signals covered by solvent residual peak*); HRMS: calcd. for C_21_H_21_Cl_2_NO_3_ 405.0898, found 405.0879.

*1-Butyl-3,5-dihydroxy-5-(3-methoxybenzyl)-4-(3-methoxyphenyl)-1H-pyrrol-2(5H)-one* (**10c**). ^1^H-NMR (DMSO-*d_6_*) δ 0.92 (t, *J* = 7.3 Hz, 3H), 1.25–1.40 (m, 2H), 1.55–1.75 (m, 2H), 3.16 (d, *J* = 13.7 Hz, 1H), 3.24 (d, *J* = 13.7 Hz, 1H), 3.36 (t, *J* = 7.5 Hz, 2H), 3.53 (s, 3H), 3.76 (s, 3H), 6.21 (m, 1H), 6.32 (d, *J* = 7.7 Hz, 1H), 6.50 (s, 1H), 6.64 (dd, *J* = 7.9, 2.2 Hz, 1H), 6.87 (dd, *J* = 7.5, 2.4 Hz, 1H), 7.00 (t, *J* = 7.8 Hz, 1H), 7.36 (t, *J* = 8.0 Hz, 1H), 7.58 (m, 1H), 10.10 (s, 1H); ^13^C-NMR (DMSO-*d_6_*) δ 164.38, 158.99, 158.37, 144.64, 136.84, 133.99, 129.28, 128.48, 121.75, 119.97, 118.44, 114.62, 113.17, 112.22, 111.81, 90.47, 54.92, 54.55, 41.06, 30.82, 20.07, 13.84 (one signal covered by residual solvent peak); HRMS: calcd. for C_23_H_27_NO_5_ 397.1889, found 397.1881.

*1,5-Dibenzyl-3,5-dihydroxy-4-phenyl-1H-pyrrol-2(5H)-one* (**10d**). ^1^H-NMR (DMSO-*d_6_*) δ 3.23 (d, *J* = 12.3 Hz, 1H), 3.33 (d, *J* = 12.3 Hz, 1H), 4.54 (d, *J* = 15.7 Hz, 1H), 4.74 (d, *J* = 15.7 Hz, 1H), 6.59 (dd, *J* = 7.7, 1.6 Hz, 2H), 6.70 (s, 1H), 6.98–7.12 (m, 3H), 7.19–7.36 (m, 4H), 7.39–7.52 (m, 4H), 7.99–8.07 (m, 2H), 10.13 (s, 1H); ^13^C-NMR (DMSO-*d_6_*) δ 164.85, 144.30, 138.70, 135.19, 132.74, 129.45, 128.31, 128.08, 127.98, 127.50, 126.71, 126.64, 126.44, 119.18, 90.62, 42.29, 41.56 (two C-H aromatic signals overlapped); HRMS: calcd. for C_24_H_21_NO_3_ 371.1521, found 371.1515.

*1-Benzyl-5-(4-chlorobenzyl)-4-(4-chlorophenyl)-3,5-dihydroxy-1H-pyrrol-2(5H)-one* (**10e**). ^1^H-NMR (DMSO-*d_6_*) δ 3.19 (d, *J* = 13.6 Hz, 1H), 3.35 (d, *J* = 13.6 Hz, 1H), 4.54 (d, *J* = 15.6 Hz, 1H), 4.74 (d, *J* = 15.6 Hz, 1H), 6.59 (d, *J* = 8.4 Hz, 2H), 6.81 (s, 1H), 7.11 (d, *J* = 8.3 Hz, 2H), 7.20–7.35 (m, 3H), 7.43 (d, *J* = 7.2 Hz, 2H), 7.52 (d, *J* = 8.7 Hz, 2H), 8.01 (d, *J* = 8.7 Hz, 2H), 10.47 (s, 1H); ^13^C-NMR (DMSO-*d_6_*) δ 164.52, 144.88, 138.50, 134.08, 131.34, 131.23, 131.17, 131.07, 129.06, 128.44, 128.07, 128.02, 127.50, 126.71, 117.95, 90.29, 42.23, 40.80; HRMS: calcd. for C_24_H_19_Cl_2_NO_3_ 439.0742, found 439.0749.

*1-Benzyl-3,5-dihydroxy-5-(3-methoxybenzyl)-4-(3-methoxyphenyl)-1H-pyrrol-2(5H)-one* (**10f**). ^1^H-NMR (DMSO-*d_6_*) δ 3.20 (d, *J* = 13.8 Hz, 1H), 3.30 (d, *J* = 13.8 Hz, 1H), 3.51 (s, 3H), 3.76 (s, 3H), 4.50 (d, *J* = 15.7 Hz, 1H), 4.70 (d, *J* = 15.7 Hz, 1H), 6.17 (br s, 1H), 6.22 (d, *J* = 7.6 Hz, 1H), 6.63 (dd, *J* = 7.9, 2.2 Hz, 1H), 6.69 (s, 1H), 6.88 (dd, *J* = 7.8, 2.2 Hz, 1H), 6.95 (t, *J* = 7.3 Hz, 1H), 7.18–7.46 (m, 6H), 7.57–7.63 (m, 2H), 10.19 (s, 1H); ^13^C-NMR (DMSO-*d_6_*) δ 164.80, 159.00, 158.38, 144.51, 138.60, 136.66, 133.95, 129.30, 128.44, 128.11, 127.97, 126.65, 121.85, 120.10, 119.09, 114.60, 113.25, 112.39, 111.99, 90.58, 54.94, 54.58, 42.28, 41.54; HRMS: calcd. for C_26_H_25_NO_5_ 431.1733, found 431.1748.

*Benzyl 3-(2-benzyl-2,4-dihydroxy-5-oxo-3-phenyl-2,5-dihydro-1H-pyrrol-1-yl)propanoate* (**10g**). ^1^H-NMR (CDCl_3_) δ 1.40–1.60 (br s, 1H), 2.72 (dt, *J* = 16.8, 5.3 Hz, 1H), 3.14 (ddd, *J* = 16.5, 8.8, 5.9 Hz, 1H), 3.25 (d, *J* =13.8 Hz, 1H), 3.39 (d, *J* = 13.8 Hz, 1H), 3.65–3.85 (m, 2H), 4.14 (br s, 1H), 5.06 (d, *J* = 12.3 Hz, 1H), 5.12 (d, *J* = 12.3 Hz, 1H), 6.70–6.75 (m, 2H), 7.05–7.15 (m, 3H), 7.25–7.35 (m, 6H), 7.45 (t, *J* = 7.6 Hz, 2H), 8.02 (d, *J* = 7.4 Hz, 2H); ^13^C-NMR (CDCl_3_) δ 173.29, 165.56, 142.12, 135.43, 134.46, 131.51, 129.67, 128.86, 128.81, 128.73, 128.29, 128.21, 127.96, 127.28, 120.90, 92.03, 67.28, 42.25, 35.51, 32.59 (*one C-H aromatic signal overlapped*); HRMS: calcd. for C_27_H_25_NO_5_ 443.1733, found 443.1742.

*Benzyl 3-(2-(4-chlorobenzyl)-3-(4-chlorophenyl)-2,4-dihydroxy-5-oxo-2,5-dihydro-1H-pyrrol-1-yl)-propanoate* (**10h**). ^1^H-NMR (DMSO-*d_6_*) δ 2.65–2.80 (m, 2H), 3.14 (d, *J* = 13.8 Hz, 1H), 3.30 (d, *J* = 13.8 Hz, 1H), 3.60–3.80 (m, 2H), 5.12 (s, 2H), 6.67 (d, *J* = 8.4 Hz, 2H), 6.70 (s, 1H), 7.14 (d, *J* = 8.4 Hz, 2H), 7.29–7.44 (m, 5H), 7.49 (d, *J* = 8.8 Hz, 2H), 7.98 (d, *J* = 8.7 Hz, 2H), 10.5 (s, 1H); ^13^C-NMR (DMSO-*d_6_*) δ 170.94, 164.31, 144.72, 136.02, 134.06, 131.29, 131.15, 131.13, 131.03, 128.92, 128.40, 128.11, 128.02, 127.93, 127.57, 117.98, 90.16, 65.68, 34.46, 33.08; HRMS: calcd. for C_27_H_23_Cl_2_NO_5_ 511.0953, found 511.0955.

*Benzyl 3-(2,4-dihydroxy-2-(3-methoxybenzyl)-3-(3-methoxyphenyl)-5-oxo-2,5-dihydro-1H-pyrrol-1-yl)-propanoate* (**10i**). ^1^H-NMR (CDCl_3_) δ 2.70 (dt, *J* = 16.6, 5.2 Hz, 1H), 3.05–3.15 (m, 1H), 3.22 (d, *J* = 14.0 Hz, 1H), 3.40 (d, *J* = 14.0 Hz, 1H), 3.57 (s, 3H), 3.65–3.85 (m, 2H), 3.84 (s, 3H), 4.11 (br s, 1H), 5.06 (d, *J* = 12.0 Hz, 1H), 5.12 (d, *J* = 12.0 Hz, 1H), 6.24 (s, 1H), 6.37 (d, *J* = 7.6 Hz, 1H), 6.65 (dd, *J* = 8.2, 2.1 Hz, 1H), 6.83–6.92 (m, 1H), 7.00 (t, *J* = 7.9 Hz, 1H), 7.24–7.41 (m, 6H), 7.56–7.67 (m, 2H); ^13^C NMR (CDCl_3_) δ 173.27, 165.52, 159.80, 159.24, 142.45, 135.90, 135.45, 132.81, 129.76, 129.16, 128.84, 128.71, 128.70, 122.00, 120.90, 120.74, 114.92, 113.78, 113.55, 113.35, 91.97, 67.26, 55.47, 55.17, 42.18, 35.48, 32.62; HRMS: calcd. for C_29_H_29_NO_7_ 503.1944, found 503.1962.

## 4. Conclusions

In conclusion we have demonstrated that the field of isocyanide-based reactions is far from being exhaustively explored and the double nucleophilic/electrophilic character can be exploited not only within the same molecule (as it happens, for example, in the Ugi and Passerini reactions) but also employing two equivalents of this reagent, as it happens in the novel multicomponent reaction reported in this paper. In addition we have shown the divergent synthesis of two classes of 5-membered nitrogen heterocycles through a unique rearragement reaction. These rearrangements are particularly interesting because they could also be applied to molecules structurally related to **3**, but not necessarily synthesized with the method described here. This aspect is particularly appealing due to the promising preliminary biological data obtained for compounds **9**.
